# Supplementary material for: Combining genome-wide polygenic scores with registry data for colorectal cancer risk-based screening
Source: Br J Cancer. 2026 Jul 15;135(8):1306–14. doi: 10.1038/s41416-026-03532-9 (PMC13642245; doi:10.1038/s41416-026-03532-9)
Supplement: Supplementary file 1 [file 41416_2026_3532_MOESM1_ESM.docx]

## Supplementary Material 1

EDTA whole blood samples were genotyped by deCODE Genetics using Illumina's Infinium Global Screening Array v1 or v3. Genotype data underwent standard quality control, including removal of duplicate samples, samples failing sex checks, and samples with missingness >5%. Variants with missingness >10% or a Hardy-Weinberg equilibrium exact test p-value < 0.00001 were removed. The QCed genotype data were imputed using deCODE genetics in-house imputation work-flow^35^ and an in-house reference panel of approximately 50,000 individuals of mixed ancestry among these around 10,800 Danish individuals.

Principal components (PCs) were estimated from the autosomal, unimputed genotype data. A random subset comprising 30% of unrelated individuals (defined as KING kinship coefficient < 0.044) was selected. Basic quality control was applied to this subset, excluding variants with >1% missingness, Hardy-Weinberg equilibrium p-values < 0.0001, and minor allele frequency <1%. Singular value decomposition (SVD) was used iteratively (up to five iterations) to remove individuals most dissimilar to the rest. PCs were then estimated on the remaining individuals using PLINK 2.0. Subsequently, all remaining individuals were projected into the principal component space defined by the unrelated subset.

Ancestry was estimated using principal component analysis (PCA) on unrelated individuals, with related individuals and 1000 Genomes Project (1KG) samples projected into the same space. To enhance clustering, Uniform Manifold Approximation and Projection (UMAP) was applied to the PCA results. Individuals genetically similar to 1KG Europeans were assigned to the “EUR” cluster using Mahalanobis distance (5 standard deviations (SD)). The same approach was applied to other major ancestry groups (African, South Asian, East Asian, and Admixed American), with genetic similarity defined as within three SDs of the Mahalanobis distance to the respective 1KG reference panel. Individuals not genetically aligned with any 1KG ancestry cluster were categorized as admixed. Finally, individuals with both parents born in Greenland who did not cluster with EUR were assigned Greenlandic (GRL) ancestry.

The first two PCs, colored by genetic ancestry, are shown in Figure 1, and the variance explained by the first nine PCs is visualized in Figure 2.

**Figure 1.** PC1 and PC2 colored by genetic ancestry

**Figure 2.** Variance explained by the first nine PCs

## Supplementary Material 2

**Method**

We conducted a sensitivity analysis restricted to individuals of European ancestry. Here the PRS calculated as the weighted sum of 203 significant CRC-associated SNPs, was compared with PRS estimates obtained using the LDpred2-auto model to assess the extent to which a genome-wide PRS approach improves the results.

The genome-wide PRS was estimated by first filtering the genotype data to variants present in the HapMap3+ marker set using PLINK2. These variants were combined into a genome-wide dataset, and missing genotypes were imputed by randomly drawing from a multinomial distribution. The genome-wide linkage disequilibrium (LD) matrix was constructed using the HapMap3+ LD matrices provided by LDpred2, downloaded from: <https://drive.google.com/uc?export=download&id=17dyKGA2PZjMsivlYb_AjDmuZjM1RIGvs>. 
Summary statistics ([<https://www.ebi.ac.uk/gwas/studies/GCST90255675>)](https://www.pgscatalog.org/score/PGS003979/) from a GWAS conducted exclusively in individuals of European ancestry were used in combination with the SNPs available in our dataset, resulting in 1,184,777 SNPs included in the LDpred2 analysis. LDpred2-auto was run on these marker SNPs with maximum likelihood estimation turned off to ensure convergence, and 20 chains with different initial values of *p* were used. Of these, 19 out of 20 chains converged according to the quality control criteria described for LDpred2 and were used to estimate the effect sizes (β-values). These were subsequently multiplied by the genotype matrix to generate PRS scores for our cohort.

**Results**

The population-recalibrated cumulative incidence analysis restricted to individuals of European ancestry showed that both the PRS estimated from 203 SNPs and the genome-wide PRS can identify individuals at both higher and lower risk for both men and women. However, the genome-wide PRS showed stronger stratification, with higher risk estimates in the ≥99th percentile (women: 11.52% (95% CI: 9.35-14.33) vs 10.37% (8.30-13.14); men: 14.74% (11.99-17.68) vs 12.73% (10.27-15.43)) and lower estimates in the bottom percentile (women: 2.29% (1.96-2.64] vs 2.55% (2.20-2.92); men: 2.86% (2.51-3.25) vs 3.12% (2.75-3.54)) compared with the 203-SNP PRS. Similar patterns were observed across intermediate groups, indicating improved separation of risk distributions (Supplementary Material 2: Figure 1 and Table 1).

Similar patterns were observed in the analysis exploring the association between PRS and lifetime CRC risk across tumor characteristics, where the genome-wide PRS provided stronger risk stratification with clearer separation between PRS groups (Supplementary Material 2: Figure 2).

The CRC and adenoma risk prediction analysis showed that the PRS estimated from 203 SNPs and the genome-wide PRS perform very similarly, with only small differences in all three prediction analyses. The genome-wide PRS showed slightly better predictions in prediction analyses 2 and 3 but did not outperform the 203-SNP PRS in analysis 1 (Supplementary Material 2: Figure 3 and Table 2).

In analysis 1, compared to the baseline + FIT model (median C-index=0.770, IQR=0.068), adding the PRS from 203 SNPs improves prediction slightly more than adding the genome-wide PRS (median C-index=0.792 vs. 0.776, IQR=0.068 vs. 0.069).

In analysis 2, compared to the baseline + FIT model (median AUC = 0.556, IQR = 0.012), adding the genome-wide PRS improves prediction slightly more than adding the PRS from 203 SNPs (median AUC = 0.586 vs. 0.576, IQR = 0.018 vs. 0.012).

In analysis 3, compared to the baseline + FIT model (median AUC = 0.572, IQR = 0.056), adding the genome‑wide PRS again yields a slightly larger improvement than adding the PRS from 203 SNPs (median AUC = 0.583 vs. 0.578, IQR = 0.079 vs. 0.045).

**Table 1. Population-recalibrated cumulative incidence estimates at age 80 for men and woman stratified using the PRS from 203 SNPs and the genome-wide PRS.**

| **PRS percentile** | **Woman** | | **Men** | |
| --- | --- | --- | --- | --- |
|  | **PRS from 203 SNPs**  CI (95%CI) | **Genome-wide PRS**  CI (95%CI) | **PRS from 203 SNPs**  CI (95%CI) | **Genome-wide PRS**  CI (95%CI) |
| PRS≥99% | 10.37 (8.30, 13.14) | 11.52 (9.35,14.33) | 12.73 (10.27,15.43) | 14.74 (11.99,17.68) |
| PRS 80–99% | 6.66 (5.87, 7.44) | 7.09 (6.26, 7.90) | 8.75 (7.93, 9.66) | 9.49 (8.61, 10.47) |
| PRS 20–80% | 4.24 (3,79, 4.69) | 4.18 (3,73, 4.61) | 5.81 (5.30, 6.43) | 5.62 (5.12, 6.22) |
| PRS 0–20% | 2.55 (2.20, 2.92) | 2.29 (1.96, 2.64) | 3.12 (2.75, 3.54) | 2.86 (2.51, 3.25) |

**Figure 1. Cumulative incidence for lifetime risk of CRC by PRS for European individuals and two PRS methods*.*** Cumulative incidence with 95% confidence bands for lifetime risk of CRC across PRS groupings. A) and B show PRS estimated in individuals of European ancestry based on 203 genome-wide significant CRC-associated SNPs for females and males, respectively. C) and D) show genome-wide PRS estimated in individuals of European ancestry for females and males, respectively.

**Figure 2. Cause-specific hazard ratios (HRs) with 95% CIs for lifetime CRC risk and two PRS methods across PRS groups**. A) PRS based on 203 genome-wide significant CRC-associated SNPs for European individuals. B) Genome-wide PRS for European individuals. HRs above 4 are truncated. AC: adenocarcinoma; dMMR: deficient mismatch repair; pMMR: proficient mismatch repair.

**Table 2. Performance of models assessing the contribution of baseline variables, FIT, and PRS, individually and in combination, across three analyses and for two PRS methods**

| **Analysis 1: Risk of adenoma or CRC from the time of first screening** | | | | |
| --- | --- | --- | --- | --- |
|  | **PRS from 203 SNPs** | | **Genome-wide PRS** | |
| **Model** | **Median C-index** | **IQR** | **Median C-index** | **IQR** |
| Baseline | 0.601 | 0.119 | 0.601 | 0.119 |
| Baseline + PRS | 0.655 | 0.064 | 0.627 | 0.090 |
| Baseline + FIT | 0.770 | 0.068 | 0.770 | 0.068 |
| Baseline + PRS + FIT | 0.792 | 0.068 | 0.776 | 0.069 |
| **Analysis 2: Colonoscopy outcome among FIT-positive participants** | | | | |
|  | **PRS from 203 SNPs** | | **Genome-wide PRS** | |
| **Model** | **Median AUC** | **IQR** | **Median AUC** | **IQR** |
| Baseline | 0.546 | 0.015 | 0.546 | 0.015 |
| Baseline + PRS | 0.570 | 0.014 | 0.582 | 0.019 |
| Baseline + FIT | 0.556 | 0.012 | 0.556 | 0.012 |
| Baseline + PRS + FIT | 0.576 | 0.012 | 0.586 | 0.018 |
| **Analysis 3 - Adenoma or CRC within two years following a negative FIT** | | | | |
|  | **PRS from 203 SNPs** | | **Genome-wide PRS** | |
| **Model** | **Median AUC** | **IQR** | **Median AUC** | **IQR** |
| Baseline | 0.544 | 0.032 | 0.544 | 0.032 |
| Baseline + PRS | 0.558 | 0.027 | 0.563 | 0.051 |
| Baseline + FIT | 0.572 | 0.056 | 0.572 | 0.056 |
| Baseline + PRS + FIT | 0.578 | 0.045 | 0.583 | 0.079 |

**Figure 3. Boxplots showing fold-wise performance of models assessing the contribution of baseline variables, FIT, and PRS, individually and in combination, across three analyses.** A) PRS based on 203 genome-wide significant CRC-associated SNPs for European individuals. B) Genome-wide PRS for European individuals. For both A) and B) three analyses were conducted: 1) Prediction of adenoma or CRC at first screening using Cox proportional hazards models, evaluated by the C-index. 2) Prediction of negative colonoscopy outcome among FIT-positive participants using logistic regression, evaluated by AUC. 3) Prediction of adenoma or CRC within two years after a negative FIT result using logistic regression, evaluated by AUC. Baseline models included age, sex, PC1-PC4, and genotyping chip.

## Supplementary Material 3

**Method**

Linear and non-linear association between PRS and lifetime risk of CRC were evaluated for Cox proportional hazards models. Thus, follow-up began at the entry date 1978-10-01 (In 1998 ICD-O coding was introduced in CAR and DNPR achieved complete nationwide coverage) or at birth for individuals born after that date. For cases, follow-up ended at age of first CRC diagnosis. For controls, follow-up ended at age of death from a cause other than CRC, age at last record in the registries or age 80. All models included left truncation at a CRC-relevant entry date (listed in Supplementary Table S1) to avoid immortal time bias. Each model was adjusted for the first four principal components, birth year, and chip version. First a linear model was made. Secondly, cubic splines models were evaluated with 3-7 knots, this is an example of a 3-knot model. For all models Akaike Information Criterion (AIC) was estimated, asymptotic likelihood ratio test (LRT) with a chi-square distribution, and visual inspection was used to compare the linear and the spline model.

**Results**

Linear and spline-based Cox models (with 3 to 7 knots) were fitted to the data to examine if there were any natural cutoffs for PRS group definitions in the associations between PRS and lifetime risk of CRC. Estimation of each models AIC showed that the spline model with 3 knots had the lowest AIC (197195.1), see Table 1. This was supported by the LRT, which indicated that the 3-knot spline model was significantly better than the linear model (p = 0.0001), while models with more than three knots did not provide a significantly better fit. However, visual inspection of the 3-knot spline model compared to the linear model revealed only minor differences in the fitted curves (see Figure 1). Thus, there were no clear clinical advantages to choosing the more complex spline model over the linear model. Furthermore, the wider CIs at the extremes of the PRS distribution reflect the limited number of individuals with very low or very high PRS, raising further questions about the robustness of the spline model in these regions. Consequently, the linear model was selected for subsequent analyses. As no natural cutoffs for PRS groupings emerged from the model, individuals were categorized into PRS groups as follows: <1%, 1-20%, 20-80% (reference), 80-99% or ≥99%. These groupings were aligned with those used in Tamlander et al. 2024^23^ to enable direct comparison.

**Table 1. Performance metrics of linear and spline-based Cox models**

| **Model** | **Knots** | **AIC** | **LRT** |
| --- | --- | --- | --- |
| **Linear** | - | 197208.0 |  |
| **Spline** | **3** | **197195.1** | **0.0001** |
|  | 4 | 197197.1 | 0.75 |
|  | 5 | 197196.9 | 0.15 |
|  | 6 | 197199.1 | 0.64 |
|  | 7 | 197200.4 | 0.40 |

**Figure 1.** Log hazard ratios (HR) with 95% CIs as a function of standardized PRS, estimated using both linear and spline-based (3-knot) Cox proportional hazards models for men and women.

## Supplementary Material 4

A subset of individuals was linked to DDSCE, and three analyses were conducted to assess whether the addition of PRS to FIT, compared with FIT alone, enhanced the performance of:

1. Prediction of adenoma or CRC risk from the time of an individual’s first screening participation.
2. Prediction of colonoscopy outcomes (positive vs. negative findings) among participants with a positive FIT.
3. Prediction of adenoma or CRC within two years following a negative FIT result.

In all three analyses model performance was evaluated for four distinct predictor sets: (1) a baseline set including age, sex, principal components 1-4 (PC1-PC4), and genotyping chip; (2) baseline plus PRS; (3) baseline plus FIT value; and (4) baseline combined with both PRS and FIT. In all analysis FIT was included as a continuous variable and linear prediction models were chosen due to the limited sample sizes.

|  | **FIT selection** | **Method** | **Performance metric** |
| --- | --- | --- | --- |
| **Analysis 1** | **First FIT test.** Follow-up began on the date of the first FIT and ended at the earliest occurrence of CRC/adenoma diagnosis, death, or censoring. | Cox proportional hazards models | C-index |
| **Analysis 2** | **First positive FIT with follow-up colonoscopy.** Cases were participants with a positive colonoscopy result, while controls were participants with a negative colonoscopy result. | Logistic regression | AUC |
| **Analysis 3** | **Case–control matched FIT.** For cases, the FIT administered closest to the time of diagnosis was selected. For controls with multiple FITs, the FIT taken at the age corresponding to the matched case was used. | Logistic regression | AUC |

**Analysis 1** assessed CRC or adenoma risk prediction using Cox proportional hazards models. The cohort included participants aged 56 years or younger at the time of their first FIT test. Time-to-event modeling was conducted using the coxph() function from the survival package. Follow-up time began at the date of the first FIT and ended at the earliest of CRC/adenoma diagnosis, death, or censoring date. 10-fold cross-validation was performed using the survcompare package, and predictive accuracy was evaluated using c-index.

For **Analyses 2 and 3**, logistic regression models were fitted using the glm() function from base R. 10-fold cross-validation was performed using the caret package, and predictive accuracy was assessed using the AUC computed with the pROC package.

**Analysis 2** focused on participants with a positive FIT result and a follow-up colonoscopy. If a participant had more than one positive FIT result the first was used. Cases were defined as participants with a positive colonoscopy, while controls included those with a negative colonoscopy result. Participants without colonoscopy data were excluded.

**Analysis 3** included participants with negative FIT results and no prior diagnosis of adenoma or CRC. Cases were defined as individuals diagnosed with adenoma or CRC within two years following a negative FIT result. Controls were selected from a pool of eligible participants who (i) had a negative FIT and no prior diagnosis of CRC or adenoma, and (ii) were not diagnosed with adenoma, CRC or died within the two years following the negative FIT. Three controls were matched to each case from this pool based on sex and age at the time of the FIT test. For cases, the FIT administered closest to the time of diagnosis was selected. For controls with multiple FITs, the FIT taken at the age corresponding to the matched case was used. Participants with an adenoma or CRC diagnosis prior to all FIT tests were excluded.

## Supplementary Tables

**Table S1.**

| **Outcome** | **Analysis** | **Left truncation date** | **Argument** |
| --- | --- | --- | --- |
| **CRC** | Cox proportional hazards models and Cumulative incidence models | 1978-01-01 | CAR was established in 1942, but data quality improved in 1978 because ICD-O coding was introduced. |
| **Adenoma** | Cox proportional hazards models and Cumulative incidence models | 1978-01-01 | DNPR achieved complete nationwide coverage in 1978. |
| **CRC histology** | Cause-specific Cox proportional hazards models | 2009-10-01 | Data on histology have been available in DCCG since this date. |
| **CRC MMR status** | Cause-specific Cox proportional hazards models | 2009-10-01 | Data on MMR protein expression have been available in DCCG since this date. |
| **CRC site** | Cause-specific Cox proportional hazards models | 2001-07-01 | Data on tumor site have been available in DCCG since this date |

**Table S2.**

| **Characteristic** | **European genetic ancestry**  N=102,165 | | | **Non-European genetic ancestry**  N=10,039 | | |
| --- | --- | --- | --- | --- | --- | --- |
|  | **Colon (C18,26)** N = 5,904^1^ | **Rectum (C19-20)** N = 2,609^1^ | **Control** N = 93,652^1^ | **Colon (C18,26)** N = 494^1^ | **Rectum (C19-20)** N = 239^1^ | **Control** N = 9,306^1^ |
| **Gender** |  |  |  |  |  |  |
| Female | 2,840 (48%) | 925 (35%) | 46,780 (50%) | 233 (47%) | 100 (42%) | 4,527 (49%) |
| Male | 3,064 (52%) | 1,684 (65%) | 46,872 (50%) | 261 (53%) | 139 (58%) | 4,779 (51%) |
| **Birth year** | 1939 (1932, 1947) | 1941 (1933, 1949) | 1942 (1932, 1951) | 1940 (1932, 1951) | 1943 (1935, 1952) | 1944 (1934, 1957) |
| **Year of diagnosis** | 2014 (2010, 2016) | 2013 (2008, 2015) |  | 2014 (2010, 2016) | 2013 (2009, 2015) |  |
| **CRC site** |  |  |  |  |  |  |
| Proximal colon | 1,272 (31%) | 0 (0%) |  | 114 (33%) | 0 (0%) |  |
| Distal colon | 2,132 (52%) | 0 (0%) |  | 177 (51%) | 0 (0%) |  |
| Unspecified colon | 664 (16%) | 0 (0%) |  | 53 (15%) | 0 (0%) |  |
| Rectum | 0 (0%) | 2,219 (100%) |  | 0 (0%) | 193 (100%) |  |
| Unknown | 1,835 | 390 |  | 150 | 46 |  |
| **ECOG**^2^ **Performance Status** |  |  |  |  |  |  |
| 0 | 1,199 (45%) | 536 (53%) |  | 87 (40%) | 48 (57%) |  |
| 1 | 804 (30%) | 277 (27%) |  | 79 (37%) | 21 (25%) |  |
| 2 | 438 (17%) | 127 (13%) |  | 39 (18%) | 10 (12%) |  |
| +3 | 206 (7.8%) | 68 (6.7%) |  | 11 (5.1%) | 5 (6.0%) |  |
| Unknown | 3,257 | 1,601 |  | 87 (40%) | 48 (57%) |  |
| **Histology** |  |  |  |  |  |  |
| Adenocarcinoma | 2,409 (41%) | 1,202 (46%) |  | 203 (41%) | 100 (42%) |  |
| Poorly differentiated adenocarcinoma | 294 (5.0%) | 51 (2.0% |  | 23 (4.7%) | 5 (2.1%) |  |
| Mucinous adenocarcinoma | 329 (5.6%) | 76 (2.9%) |  | 35 (7.1%) | 5 (2.1%) |  |
| Unknown or carcinoma (Medullary, Signet ring cell, Undifferentiated) | 2,872 (49%) | 1,280 (49%) |  | 233 (47%) | 129 (54%) |  |
| **DNA mismatch repair (MMR)** |  |  |  |  |  |  |
| Deficient (dMMR) | 557 (19%) | 19 (1.5%) |  | 46 (19%) | <5 |  |
| Proficient (pMMR) | 2,390 (81%) | 1,210 (98%) |  | 196 (81%) | 99 (99%) |  |
| Unknown | 2,957 | 1,380 |  | 252 | 139 |  |
| **Adenoma diagnosis** |  |  |  |  |  |  |
| Adenoma | 1,165 (20%) | 453 (17%) | 6,712 (7.2%) | 75 (15%) | 32 (13%) | 558 (6.0%) |
| No adenoma | 4,739 (80%) | 2,156 (83%) | 86,940 (93%) | 419 (85%) | 207 (87%) | 8,748 (94%) |
| **PRS strata** |  |  |  |  |  |  |
| 0-20 | 680 (12%) | 251 (9.6%) | 19,209 (21%) | 69 (14%) | 18 (7.5%) | 2,214 (24%) |
| 20-80 | 3,484 (59%) | 1,533 (59%) | 56,603 (60%) | 283 (57%) | 146 (61%) | 5,273 (57%) |
| 80-99 | 1,627 (28%) | 763 (29%) | 17,021 (18%) | 133 (27%) | 71 (30%) | 1,703 (18%) |
| ≥99 | 113 (1.9%) | 62 (2.4%) | 819 (0.9%) | 9 (1.8%) | <5 | 116 (1.2%) |
|  |  |  |  |  |  |  |

[1] n (%); Median (Q1, Q3), [2] ECOG = Eastern Cooperative Oncology Group.

**Table S3.**

| **Age** | **Female** | | **Male** | |
| --- | --- | --- | --- | --- |
|  | **Population incidence CRC^1^** | **Age (years) at incidence in this cohort** | **Population incidence CRC^1^** | **Age (years) at incidence in this cohort** |
| **45 years** | 0.10% |  | 0.09% |  |
| PRS≥99% |  | 39 |  | 39 |
| PRS 80–99% |  | 42 |  | 42 |
| PRS 20–80% |  | 45 |  | 45 |
| PRS 0–20% |  | 48 |  | 49 |
| **50 years** | 0.21% |  | 0.20% |  |
| PRS≥99% |  | 44 |  | 45 |
| PRS 80–99% |  | 47 |  | 47 |
| PRS 20–80% |  | 50 |  | 50 |
| PRS 0–20% |  | 54 |  | 54 |
| **55 years** | 0.41% |  | 0.43% |  |
| PRS≥99% |  | 49 |  | 50 |
| PRS 80–99% |  | 52 |  | 52 |
| PRS 20–80% |  | 55 |  | 55 |
| PRS 0–20% |  | 59 |  | 59 |
| **60 years** | 0.79% |  | 0.87% |  |
| PRS≥99% |  | 53 |  | 54 |
| PRS 80–99% |  | 56 |  | 56 |
| PRS 20–80% |  | 60 |  | 60 |
| PRS 0–20% |  | 65 |  | 65 |

## [1] Incidence estimates for Denmark from Global Burden of Disease (GBD) 2021 data.

**Table S4.**

| **Characteristic** | **MMR deficient** N = 623 | **MMR proficient** N = 3,895 |
| --- | --- | --- |
| **Diagnosis group** |  |  |
| Colon (C18,26) | 603 (97%) | 2,586 (66%) |
| Rectum (C19-20) | 20 (3%) | 1,309 (34%) |
| **Gender** |  |  |
| Female | 408 (65%) | 1,493 (38%) |
| Male | 215 (35%) | 2,402 (62%) |
| **Birth year** | 1941 (1936, 1946) | 1944 (1937, 1951) |
| **Year of diagnosis** | 2016 (2013, 2018) | 2015 (2013, 2017) |
| **CRC site** |  |  |
| Distal colon | 63 (14%) | 1,318 (38%) |
| Proximal colon | 303 (68%) | 575 (17%) |
| Rectum | 20 (4.5%) | 1,309 (38%) |
| Unspecified colon | 61 (14%) | 236 (7%) |
| Unknown | 176 | 457 |
| **ECOG Performance Status^2^** |  |  |
| 0 | 179 (42%) | 1,372 (56%) |
| 1 | 151 (35%) | 686 (28%) |
| 2 | 71 (17%) | 270 (11%) |
| +3 | 29 (7%) | 118 (5%) |
| Unknown | 193 | 1,449 |
| **Histology** |  |  |
| Adenocarcinoma | 301 (48%) | 3,339 (86%) |
| Unknown or carcinoma (Medullary, Signet ring cell, Undifferentiated) | 67 (11%) | 37 (1%) |
| Poorly differentiated adenocarcinoma | 138 (22%) | 212 (5%) |
| Mucinous adenocarcinoma | 117 (19%) | 307 (8%) |
| **Adenoma diagnosis** |  |  |
| Adenoma | 119 (19%) | 762 (20%) |
| No adenoma | 504 (81%) | 3,133 (80%) |
| **Ancestry** |  |  |
| European | 576 (92%) | 3,600 (92%) |
| Non-European | 47 (8%) | 295 (8%) |
| **PRS strata** |  |  |
| 0-20 | 105 (17%) | 380 (10%) |
| 20-80 | 381 (61%) | 2,312 (59%) |
| 80-99 | 129 (21%) | 1,123 (29%) |
| ≥99 | 8 (1%) | 80 (2%) |

[1] n (%); Median (Q1, Q3), [2] ECOG = Eastern Cooperative Oncology Group

**Table S5.**

| **Analysis 1: Risk of adenoma or CRC from the time of first screening** | | |
| --- | --- | --- |
| **Model** | **Median C-index** | **IQR** |
| Baseline | 0.63 | 0.07 |
| Baseline + PRS | 0.68 | 0.09 |
| Baseline + FIT | 0.78 | 0.02 |
| Baseline + PRS + FIT | 0.79 | 0.05 |
| **Analysis 2: Colonoscopy outcome among FIT-positive participants** | | |
| **Model** | **Median AUC** | **IQR** |
| Baseline | 0.55 | 0.01 |
| Baseline + PRS | 0.58 | 0.02 |
| Baseline + FIT | 0.56 | 0.01 |
| Baseline + PRS + FIT | 0.58 | 0.02 |
| **Analysis 3 - Adenoma or CRC within two years following a negative FIT** | | |
| **Model** | **Median AUC** | **IQR** |
| Baseline | 0.55 | 0.03 |
| Baseline + PRS | 0.57 | 0.03 |
| Baseline + FIT | 0.59 | 0.03 |
| Baseline + PRS + FIT | 0.60 | 0.02 |

## Supplementary Figures

**Figure S1.** Cumulative incidence of lifetime colorectal cancer risk, stratified by European and non-European ancestry. Note: The cumulative incidence has not been recalibrated to the Danish population, as this would require the assumption that incidence, prevalence, and mortality estimates are directly comparable across the two groupings, which was not deemed appropriate.

**Figure S2.** Cumulative incidence of lifetime colorectal cancer risk across PRS groups, stratified by European and non-European ancestry.

**Figure S3.** HR with 95% CIs for lifetime risk of colorectal cancer and adenoma across PRS groupings, stratified by sex.

**Figure S4.** Cumulative incidence with 95% CIs of lifetime colorectal cancer risk across PRS groupings, stratified by MMR status. Note: The cumulative incidence has not been recalibrated to the Danish population, as this would require the assumption that incidence, prevalence, and mortality estimates are directly comparable across the two groupings, which was not deemed appropriate.
